# Midwifery education, regulation and association in the Democratic Republic of Congo (DRC) – current state and challenges

**DOI:** 10.1080/16549716.2020.1717409

**Published:** 2020-01-27

**Authors:** Malin Bogren, Britou Ndela, Carla Toko, Marie Berg

**Affiliations:** a Institute of Health and Care Sciences, Sahlgrenska Academy, University of Gothenburg, Gothenburg, Sweden; b SANRU Asbl, Ville de Kinshasa, Democratic Republic of Congo

**Keywords:** Midwifery profession, health workforce, education, regulation, association, Democratic Republic of Congo, East and Southern Africa

## Abstract

**Background**: In the Democratic Republic of Congo (DRC), maternal and neonatal health outcomes are poor and delivering healthcare services of sufficient quality is a challenge as there are only 0.6 midwives, physicians, or nurses for every 1,000 inhabitants.

**Objective**: To explore the current state of the midwifery profession in the DRC and to suggest suitable strategies for increasing the quality and quantity of a highly competent midwifery health workforce in the DRC.

**Methods**: Data were collected at a workshop with 17 key persons using three questionnaires developed by the International Confederation of Midwives, and three focus group discussions. The analysis was focusing on quantitative and qualitative content.

**Results**: In DRC the midwife profession is not legislated. A midwifery association is well established, but due to a lack of resources does not function optimally. Two midwifery education programmes exist: a three-year direct-entry programme resulting in a diploma in midwifery, and a 12-month postgraduate programme for nurses resulting in a certificate in midwifery. Neither of the programmes leads to a bachelor’s or master’s degree. At the institutions offering the midwifery programmes (n = 16), the educators’ academic qualifications are lower than required and there is a lack of teaching and training equipment for meeting the education needs.

**Conclusions**: The Sustainable Development Goal on health, and specifically the health of mother and newborn, will be difficult to meet in the DRC. We therefore suggest that (i) the midwifery education programmes be improved to meet international standards; (ii) these programmes be designed in a way that allows for an academic degree at either the bachelor’s or master’s level; (iii) the competence level of the midwifery educators be increased; and, most crucially, (iv) a regulatory structure be formed that legislates and regulates the midwifery profession and its autonomous practice.

## Background

A strong professional workforce of midwives, educated and regulated to international standards and fully integrated into the health system [1,2], is critical for delivering high-quality sexual, reproductive, maternal, newborn, and adolescent care [3,4]. Scaling up the quality and quantity of the midwifery workforce is one important strategy for expanding such care [3–6]. However, ensuring the provision of midwifery care to meet the Sustainable Development Goals (SDGs) by the year 2030 [7] is a serious challenge for many health systems, especially in countries where resources are scarce, as they are in East, Central, and Southern Africa, and particularly in the Democratic Republic of Congo (DRC) [8].

In the DRC, there are major challenges in relation to the quality of healthcare services, especially for mothers and their babies. With a fertility rate of 6.6 births per woman, a contraceptive prevalence rate of 8%, a maternal mortality rate of 846 per 100,000 live births, and a neonatal mortality rate of 28 per 1,000 live births [9], and with only 0.6 midwives, physicians, or nurses for every 1,000 inhabitants [10], the challenges are huge. Without the right healthcare investments, including a highly competent health workforce, it will not be possible to meet the SDG 3 on health by 2030.

The DRC government has recognized the midwifery profession as essential to achieving better sexual and reproductive, maternal, newborn, and adolescent health outcomes [8]. In accordance with the profession theory, which defines the key elements of a profession [11–13], it is of great importance to ensure that the midwifery profession is based on a scientific body of knowledge, trained skills and an ethical code; requires a licence to practise; has autonomy; and is recognised by society [14]. The three pillars – Education, Regulation, and Association (ERA) – provide a framework to assist countries in operationalizing the process of scaling up the production of well educated, professional midwives to meet global standards, in order to ensure the provision of high-quality evidence-based midwifery care [15]. For becoming a midwife, global standards comprise two types of education: a minimum of three years for direct-entry midwifery programmes, or a 1.5-year post-nursing programme [16].

The midwifery profession, as defined by the International Confederation of Midwives (ICM) [17], is new in the DRC. During the country’s colonial period, midwifery training began at a secondary-school level and those who completed it were recognized as A3-level birth attendants. This training was abolished and was replaced by a four-year midwifery education programme, at a secondary- school level, recognized as A2-level Auxiliary Midwives. Since the country’s educational reform in 2013, a three-year midwifery education is conducted at a higher education level, which is in line with international norms and standards. Those completing the programme are recognized as A1-level midwives (personal communication with B. Nkonka, Ministry of Higher Education, and A. Mulunda, Educator at ISTM Kinshasa, 22 May 2019, and A. Kabeya, Midwifery Association, 23 May 2019).

Understanding the current state of the midwifery profession in the DRC, including its gaps and challenges, is an essential basis for identifying suitable strategies for strengthening the midwifery workforce to meet the challenges of the SDGs, especially on health. In this paper, we present the current situation of the midwifery profession in the DRC, with focus on its education, regulation, and professional association. We also give recommendations for suitable strategies for improvement useful for both DRC and other countries in similar contexts.

## Methods

In order to get an overview as well as a deeper understanding of the gaps and challenges concerning midwifery education, regulation, and professional association in the DRC, an integration of qualitative and quantitative data was analysed [18].

### Data collection and participants

Data were collected at a three-day workshop in Kinshasa in the DRC in March 2019, with 17 participants representing: the Ministry of Health (n = 5), Ministry of Higher Education (n = 3), three higher education institutes delivering education programmes for midwives (n = 3), the national midwifery association (n = 1), and a non-governmental organization (NGO) (n = 5), all key country representatives of midwifery in the DRC. The workshop had been organized by the national NGO Soins de Santé Primaires en Milieu Rural (SANRU Asbl) and was facilitated by BN, MBo, and MBe. Ethical approval for the study was given by the Research Ethics Committee at the Congo Protestant University. All participants were given oral information about the study and their rights, such as the right to refrain from answering a question or to discontinue the interview without explanation at any time.

Firstly, the ICM gap analysis questionnaires (n = 3) were used to get an overview of the situation in the DRC regarding the three pillars of education, regulation, and association for the midwifery profession. The questionnaires are based on global midwifery education and regulation standards set by the ICM [19,20], and on a capacity tool for the assessment of professional midwifery associations [21]. The questions cover the assessment of the education system for midwives with qualifications, the regulation and licensing systems for the midwife profession, and the capacity and position of the midwifery association. The structured questions in the questionnaires included answers such as ‘Yes’, ‘No,’ ‘Don’t know’ and ‘Estimated numbers’, with possibilities to elaborate through narrative responses. Two of the authors (MBo and CT) asked the questions and filled out the questionnaires. Answers related to specific competencies, the prescription and administration of drugs, and details about standards related to individual educational institutions are not presented in this paper, as these will be presented in another study on midwives’ competence requirements. For details about the questionnaires, see additional files 1, 2 and 3.

Secondly, deepened information was collected through focus group discussions (FGDs) with the workshop participants after their consent. The FGDs had three to four participants in each group representing the Ministry of Health, Ministry of Higher Education, higher education institutes delivering education programmes for midwives, and the national midwifery association. The FGDs were held in French, led by MBe and with assistance from MBo, who also made field notes. Open questions with follow-up questions were posed, focusing on the education, regulation and association of midwives in the DRC. The FGDs were audiotaped and lasted 45–60 minutes.

### Analysis

The analysis was based on basic information from the questionnaires and a qualitative analysis of the deepened information from the FGDs [18]. The audiotaped FGDs were transcribed from French into English text, which, together with the field notes, was analysed by MBo and MBe using a deductive content analysis method according to Elo Kyngäs [22]. First, repeated readings of the text was conducted to get an overall understanding of the current situation of the midwifery profession in the DRC. Next, in new text readings, meaning units were identified that grasped the essentialities and the meaning units were compared for similar content and coded. Next, the codes were sorted to either education, regulation, or association, followed by clustering into subcategories. Finally, after several refinements of the analysis, a final consensus was reached on the reported results. An example of the data analysis process is illustrated in Table 1.

**Table 1.** Examples of the data analysis process from meaning unit to category

FGD	Meaning unit translated from French to English	Code	Subcategory	Category
FGD 1	The 12-months conversion programme needs to be extended to 18 months, and it also need to be at a higher academic level.	A longer conversion Midwifery education programme is needed to be at right academic level	Low academic level and insufficient length of the midwifery education programmes	Education
FGD 2	The new midwifery conversion programme builds on the ICM criteria which gives us prerequisites to promote maternal and newborn health, but we need the education to be 18 months to fully follow the ICM standards	The conversion programme needs to be 18 months to fulfil the ICM standards		
FGD 1	Some of the clinical preceptors have too low midwifery qualification. This is a problem when educating the midwifery students.	Lack of academic qualification in midwifery preceptors	Lack of qualified midwifery educators and clinical preceptors	
FGD 2	To secure the midwifery profession is autonomous, we need qualified midwifery educators at a PhD level. There are no midwives in DRC with a PhD degree, and we have no professors who are midwives.	No midwifery educators with a PhD level		
FGD 3	I want to stress the lack of competence that the midwifery educators have. We lack educators who are midwives, with a higher academic qualification.	Lack of academic qualification in midwifery educators		

## Results

The current state of the midwifery profession in the DRC is sorted into three main categories: *Midwifery Education* comprising three subcategories: (1) Low academic level and insufficient length of the midwifery education programmes, (2) Lack of qualified midwifery educators and clinical preceptors, and (3) Lack of resources to deliver a midwifery education programme of sufficient quality; *Midwifery Regulation* comprising two subcategories: (1) No regulatory structure and (2) No systems for deployment or remuneration; and *Midwifery Association* with two subcategories: (1) An established midwifery association, well connected and accepted, and (2) Lack of resources to function optimally.

### Midwifery education

#### Low academic level and insufficient length of the midwifery education programmes

At the time of the study, there were only two official educational programmes for becoming a midwife in the DRC, both offered at the higher education level (university or similar). These comprised a three-year direct-entry programme, the minimum entrance requirement for which was completion of secondary school, and a 12-month in-service programme for nurses educated within a three-year programme at higher academic level. Neither of these programmes provided opportunities for an academic degree such as a bachelor’s or master’s. Students completing the direct-entry programme received a midwife diploma, and nurses completing the 12-month in-service education programme received a midwife certificate.

The direct-entry programme was offered at 16 higher education institutes located in 12 of the country’s 26 provinces, whereof three of the higher education institutes offered the 12-month in-service education programme. The curriculum for each of the two educational programmes consisted of 60% theory and 40% clinical practice. By 2018, 3,646 midwives had been educated within one of these two programmes. No information was available on how many midwives had been educated in each programme.

Two weaknesses were highlighted in relation to the 12-month in-service midwifery education programme. It was not placed at a higher academic level than the nursing programme (which was a compulsory prerequisite for it), and it did not correspond to the ICM-established length of a post-nursing programme:

We must strengthen the academic level of our midwifery education programme for nurses. As it is today, we conduct midwifery education at a diploma level, but we also need an education programme at a master’s degree level. … But we need the education to be 18 months to meet the ICM’s standards. Through this in-service education, it’s faster to educate midwives compared to the three-year direct-entry programme. (FGD 2) // The 12-month in-service midwifery education programme needs to be revised to meet the required 18 months as recommended by the global standards. (FGD 1)

#### Lack of qualified midwifery educators and clinical preceptors

There was lack of qualified midwifery educators and clinical preceptors to deliver the midwifery education programmes. The need to build faculty competence and capacity was stressed. The minimum academic level required for educators teaching in the midwifery programme was a bachelor’s degree. It was reported as a problem that the DRC lacked educators with academic qualifications higher than those of the students in the programme they were teaching in. Ideally, educators should have a master-level education, and there should also be someone with a PhD-level education. To increase the pedagogic competence, an exchange of teachers between different institutes/universities would be beneficial. But for this, financial support was needed:

The teachers must have the right competence to teach in the midwifery education programmes. We lose women in childbirth because the education is not adequately delivered. (FGD 1) // To improve the education, the teachers must first learn the content of the midwifery curriculum. The teachers need to first become midwives themselves before they can start teaching in the programme. It’s challenging teaching the new programme. It’s new methodology and new content. (FGD 3)

Another identified challenge was the formal education level of the clinical preceptors. Most of the midwives/nurses had the same, or lower, academic qualifications as those of the students they were supervising. A further challenge was when the clinical preceptors did not provide the same care as being educated at the institutes:

The clinical teachers at the clinical sites and the educators at the university have different levels of education. This is a challenge. It’s difficult to harmonize the different levels. The clinical teachers must have a higher educational level than the students they supervise. It becomes a problem when they don’t follow the same teaching in the clinic as at the university. The clinical preceptors need further education so they can reach the same level as the educators at the university. (FGD 3)

#### Lack of resources to deliver a midwifery education programme of sufficient quality

At the teaching institutions, there was a lack of material and infrastructure resources, and the educators had low academic qualifications. Because of the lack of premises and equipment, it was challenging for educators to follow the teaching and learning methodology suggested in the curriculum. This endangered the delivery of high-quality education and the provision of high-quality care.

The lack of material involved everything from essential resources, like teaching aids and training equipment, to more advanced supplies like electricity, water, and Internet. It also involved the provision of facilities to deliver simulation-based learning, and other facilities such as a library and computer labs. Insufficient labour and birthing beds, resuscitation materials, and medicines were other prime concerns:

The lack of electricity, water, and Internet hinders the delivery of education and the provision of adequate and correct care. (FGD 1) // The biggest challenge is the lack of teaching aids and training equipment for the simulation-based learning necessary to be well trained to care for women and newborns. (FGD 2)

We need teaching aids and equipment at the hospitals, such as delivery beds, resuscitation materials, and medicine. The midwifery schools lack the availability of a library and IT equipment, for both teachers and students. (FGD 2)

### Midwifery regulation

#### No regulatory structure

There was an officially recognized definition of a professional midwife; it was identical to the ICM definition, and the profession was included in the national health sector plan. However, there was no professional or legal regulatory structure upholding the profession. This was expressed as being a risk and a threat to societal health:

The risks involved with not having midwifery regulations are that the profession is not protected, there is no professional autonomy, and without this, the health of our society is at risk. This is why we need a law about the role and responsibility of the profession midwife. (FGD 2)

A consequence of not having the midwifery profession regulated was that there was no relationship between the education and regulation pillars, such as a national accreditation system for higher education:

We need to create an accreditation system. There must be a system for assessing the quality of the universities, so it can be assessed whether they are of adequate quality to educate midwives. (FGD 2)

With no midwifery regulation, there was no regulatory authority defining the scope of practice of the midwife consistent with the ICM definition and the scope of practice for a midwife; nor was there a philosophy or code of ethics for midwives. With no regulation in the country, it was reported that there were no criteria for registration and/licensure of the profession. As a result, nurses called themselves midwives despite having earned a nursing degree. A separate midwifery Act regulating the profession would avoid this. An Act, according to the participants, would ensure safe healthcare practice delivered from a competent and autonomous midwifery workforce:

There is no Act regulating the midwifery profession. Such an Act is needed to regulate the autonomy of the profession and define the competencies required to ensure public safety. It would also ensure that not just anybody could call themselves a midwife. This would secure the provision of high-quality care. (FGD 3)

#### No systems for deployment or remuneration

There were no national deployment or remuneration systems in place for midwives. This meant, for example, that a new midwife could be placed at a surgery or medicine ward after graduation, and not be given the opportunity to provide the service she had been educated for. Furthermore, midwives working within the profession experienced low remuneration, which contributed to a lack of motivation to remain within the profession:

They (the midwives) have begun to fight for their job security. Care during childbirth should be conducted by autonomous midwives, and nurses shouldn’t have the same role as them. A midwife must be deployed as a midwife, not as a nurse, at a surgery ward. Healthcare managers need to be informed about the different competencies between a nurse and a midwife. (FGD 1)

### Midwifery association

#### An established midwifery association, well connected and accepted

Since the year 2000, the DRC has had an established professional midwifery association, with about 1,700 members, and since 2017, it is a member of the ICM. Its role was to provide continuing professional development, undertake advocacy and negotiations with the government on educational matters such as quality standards, and address professional matters for midwives such as regulation, deployment, and working conditions:

The association is supporting the educational progression at the universities, and is working hard with the establishment of a separate profession of midwives. (FGD 1) // The problem is that we don’t have an Act. This prevents the midwifery association from being established as a professional trade union with its own number. We’re registered as an association. (FGD 2)

The midwifery association was accepted in the country and had a well-established collaboration with the government. A need was expressed for more sensitization about the profession, from community to parliament. This, as people in general do not know the difference between traditional birth attendants, nurses and midwives:

An Act needs to be advocated for, in parliament but also in society. (FGD 3) // Society doesn’t know the difference between a traditional birth attendant, nurse or a midwife; more advocacy through media is needed. (FGD 3)

Although the profession of midwife was accepted by the government, the common understanding among participants was that it was a national expectation that the midwifery association should take the lead in advocating for improved working conditions for midwives.

#### Lack of resources to function optimally

The association had their own office to support and facilitate its daily work, but lacked the necessary equipment to function optimally. Its financial resources were limited, and the staff working there were not being paid. The association’s administrative policies and procedures were in place, but lacked human resources and employment policies as well as an accounting system:

We have a small office, which we need equipment for. We must have the right economic conditions to do our work; as it is now, the office staff are working without any payment. (FGD 2)

## Discussion

Our study shows that the midwifery profession in the DRC mirrored through the profession theory [14], had not sufficiently acquired all the characteristics a profession should have: a scientific body of knowledge, trained skills and an ethical code; the requirement of a licence to practise; autonomy; and the formal recognition of society [15]. The risk of not having acquired all these characteristics can be understood as the midwifery profession in the DRC having to compete with other healthcare providers for professional jurisdiction to provide the full scope of midwifery practice. There are currently two midwifery education programmes offered at 16 institutions across DRC: one 3-year programme leading to a diploma in midwifery, and one 12-month post-nurse graduate programme leading to a certificate in midwifery, offered at three of these institutions. Neither of these programmes provided opportunities for an academic degree at the bachelor’s or master’s level. The educators’ academic qualifications were too low and there were insufficient teaching aids and training equipment for meeting the education needs. We further found that the midwifery profession is not yet legislated or regulated in the DRC. Thus, there is no logical relationship between education, regulation and practice, which according to the WHO midwifery education action framework is crucial for securing high-quality midwifery healthcare [23].

A strength was that a midwifery association was established in the DRC, and had well-established collaboration with the government as well as with the respective professional organizations for nurses and medical doctors. But the association’s functioning was limited, as it had insufficient resources to do an optimal job. Professional associations like this midwives’ association, according to scientific evidence [24,25], are important key stakeholders, and can play an important role in policy discussions related to the education, regulation, and performance of the midwifery workforce [24]. In line with the theory of profession, professional bodies (such as midwifery associations) play an important role in the promotion of professionalization, including professional interests on a broader organizational and political level [13].

### Suggested interdependent strategies to move forward in strengthening a midwifery profession in the DRC aligned with global standards

The preparation of midwife educators, and midwives informed by global standards [19,26], was a challenge. Neither of the two existing midwifery education programmes was organized so that an academic degree at a bachelor’s or master’s level could be earned. We believe it is crucial that the DRC develops the educational system for midwives. In accordance with a study about the process in Sweden when the midwifery education was transferred from diploma to postgraduate or master’s level [27], such an educational system in the DRC would lead to midwifery students there earning not only a professional diploma in midwifery but also an academic degree at postgraduate level. Thus, it would lead to opportunities for advancement in midwives’ academic career. It is argued that the academization of the midwifery profession would strengthen not only the professional and disciplinary development within the profession but also the evidence-based knowledge and skills in theory and practice [27]. Similar lessons can be learned from studies conducted in Canada [28], East Africa [29,30], Europe [31,32], South Asia [33,34], and New Zealand [35], where midwifery education has been successfully integrated within higher education and now combines academic and clinical practice and produces competent, confident midwives and educators who are able to work across the midwifery scope of practice with proper merits.

A higher academization level of the midwifery education not only places higher demands on students but also requires higher competencies in educators [27]. Midwifery educators, according to global standards, should be midwives themselves with academic qualifications at least one degree higher than those of the students in the programme they are teaching [19]. Based on our findings and the fact that quality midwifery care, provided by midwives educated to international standards, reduces maternal and newborn mortality and stillbirth rates by over 80% [3], we thus stress the importance of preparing midwifery educators’ competence informed by global standards, which includes both the clinical and theoretical competence to secure quality midwifery education and quality of care.

It is known that a legislated, regulated and licenced midwife profession, educated according to international standards, can provide high-quality, preventive, supportive, and health-promoting care to adolescents, women, and newborns [4]. As there is no existing regulatory body regulating the midwife profession in the DRC, based on the theory of profession [13] we argue that it is important that the DRC establishes a midwifery regulatory structure which legislates and regulates the profession and its autonomous practice. These recommendations are also supported by the ICM global standards of midwifery regulation [20], the Lancet series on midwifery [1,3,4], and the State of the World’s Midwifery in East & Southern Africa [8]. Regulation is built upon legislation, and sets the criteria and processes that support midwives in working autonomously within their full scope of practice, guided by the principles of a code of ethics [20]. By establishing a midwifery regulatory structure that legislates and regulates the profession and its autonomous practice, the DRC will have the same opportunities as other low- and middle-income countries [36,37] to have standards dictating who can use the title of midwife and who cannot. The fact that this implies the importance of establishing a professional and/or legal regulatory structure that upholds the profession protects the title of midwife, meaning that no other profession can use the title, and ensures the relationship between midwifery education, regulation, and practice. This, to uphold high-quality education standards, and ensure that midwives are competent and fit to practise and are accountable for the services they provide.

It is crucial, and a strength, that the midwifery association in the DRC was established and well recognized by the government. Similar recognition among midwifery associations has been identified in South Asia [38] and East and Southern Africa [8,25] as critical for the existence of the profession. However, despite governmental recognition, midwifery associations in both the DRC and other countries [37,39,40] are facing challenges in leading the way in efforts to establish standards for education, regulation, and the scope of midwifery practice, and to raise demand for midwives as a profession separate from nursing. Thus, another strategy we propose for strengthening the professional midwifery association in the DRC is to support it in leading in defining the scope of practice and competencies needed for midwives in the DRC, and to strengthen the association’s communication, advocacy, and resource mobilization planning capacity for advancing the midwifery profession. In line with the findings here, as well as in 73 midwifery associations around the world [24], the association’s involvement in midwifery workforce planning and decision-making processes was limited. Thus, the strategy is suggested in order to increase the involvement of midwives in national policy development, and in the transition towards an autonomous midwifery practice.

#### Strengths and limitations

The key strength of this study is that it is the first of its kind in the DRC. Secondly, the study was conducted by an interprofessional research team containing researchers from different disciplines from both the DRC and Sweden. A limitation is that the data were collected through self-reports by selected participants, and was not collected at each of the 16 institutes where the midwifery education is offered. Hence, it may not be representative of all relevant institutes of higher midwifery education with the respective local governments. However, trustworthiness of the data was secured by the fact that the participants were all key persons involved in strengthening midwifery education, regulation, and association at a national level. Altogether, the participants had knowledge at both a policy level as well as higher education institutes.

To our knowledge, this study is the first documentation of the midwifery profession in the DRC when it comes to education, regulation, and its professional association. The study, from the collection of information through the analysis and presentation of findings, followed a generic research design model.

An activity that we assess to be an effect of our workshop with central stakeholders is that the United Nations Population Fund in Kinshasa has arranged a workshop focusing on the midwifery education programmes, and has suggested that the 12-month postgraduate midwifery education programme be extended to 16 months. This is awaiting official adoption by the Ministry of Higher Education (personal communication with Henriette Eke, Mbula, UNFPA Midwifery Advisor 24 September 2019).

## Conclusion

Our study shows that there are two programmes in the DRC for midwifery education on the higher education level. However, the competencies of the midwifery educators and clinical preceptors are insufficient, and the institutes offering the programmes lack the necessary materials. The 12-month postgraduate programme does not fulfil the ICM criteria and is too short. Furthermore, there is lack of legal regulatory structure to uphold the midwifery profession. It is therefore not possible to ensure a high-quality workforce of midwives in DRC. In addition, with the low academic qualifications of midwifery educators and the lack of material resources for teaching, there are major challenges to meet SDG 3 on health; specifically, the health of mother and neonate will not be met. We suggest an educational system that offers midwifery education at postgraduate and master’s level, and a regulatory structure, which legislates and regulates the midwifery profession and its autonomous practice. The midwifery association is well established but needs more resources. Consideration should be given to support the midwifery association in the DRC in raising the identity of midwives in order to advance the midwifery profession.

We believe that this publication will accelerate the establishment of legislated midwives with the right education in the DRC, which in turn will contribute to reduced maternal and neonatal mortality and to improved health in mothers and newborns. Our findings form a basis for the concrete strategies that are needed.

## Data Availability

The data sets used and/or analysed during the current study are available from the corresponding author on reasonable request.

## Paper context

In DR Congo (DRC), the midwifery profession is newly introduced, and little is known about the education, and regulatory structure around it. We describe the current state of the midwifery profession in DRC, including its gaps and challenges. We also propose strategies critical for improvement for strengthening the midwifery workforce to meet the challenges of the SDGs, especially on health. These strategies can possibly be useful to other countries in similar contexts.
